# A Proposal for Six Sigma Integration for Large-Scale Production of Penicillin G and Subsequent Conversion to 6-APA

**DOI:** 10.1155/2014/413616

**Published:** 2014-02-24

**Authors:** Anirban Nandi, Sharadwata Pan, Ravichandra Potumarthi, Michael K. Danquah, Indira P. Sarethy

**Affiliations:** ^1^Department of Biotechnology, Jaypee Institute of Information Technology, Noida, Uttar Pradesh 201307, India; ^2^Department of Chemical Engineering, Indian Institute of Technology Bombay, Powai, Mumbai 400076, India; ^3^Department of Chemical Engineering, Monash University, Clayton, Victoria 3800, Australia; ^4^Department of Chemical and Petroleum Engineering, Curtin University of Technology, 98009 Miri, Sarawak, Malaysia

## Abstract

Six Sigma methodology has been successfully applied to daily operations by several leading global private firms including GE and Motorola, to leverage their net profits. Comparatively, limited studies have been conducted to find out whether this highly successful methodology can be applied to research and development (R&D). In the current study, we have reviewed and proposed a process for a probable integration of Six Sigma methodology to large-scale production of Penicillin G and its subsequent conversion to 6-aminopenicillanic acid (6-APA). It is anticipated that the important aspects of quality control and quality assurance will highly benefit from the integration of Six Sigma methodology in mass production of Penicillin G and/or its conversion to 6-APA.

## 1. Introduction

Out of any other entities in the world of pharmaceuticals, perhaps Penicillin needs no introduction. Right from the time of World War II, Penicillin, the first discovered *β*-lactam antibiotic (*β*LA), has been and still is one of the most widely used antibiotic agents, in terms of annual bulk production (~3 × 10^7^ kg/year), annual sales (~$15 billion), and market share (~65% of the total antibiotic market) [1–3]. It is no secret that the growth of many penicillin-resistant pathogens has prompted only a minor portion of penicillins to be utilized for therapeutic functions [4]. On the other hand, the majority are used as precursors for manufacturing semisynthetic penicillins (SSPs). This further boosts the effectiveness of penicillins and also widens the range of antimicrobial properties or activities [2, 5].

Production of SSPs is essentially a two-step process: first, penicillins (in bulk) are converted to 6-aminopenicillanic acid (6-APA) through a procedure mediated through chemicals or enzymes. Next, extended processing of 6-APA is achieved by using enzymes to get the final product (SSPs) [6]. Conventionally, 6-APA used to be synthesized principally by hydrolysis of penicillins using unsafe chemicals and solvents (trimethylchlorosilane, phosphorous pentachloride, and dichloromethane), at unusually low temperatures [6]. Currently, the preferred method for this is enzymatic alteration of bulk penicillins into 6-APA using penicillin acylase [4]. In the context of the current work, we will focus only on Penicillin G acylase (PGA). It is interesting to note that till date several stages for enhanced heterologous production of PGA have been proposed using recombinant DNA technology and for this* Escherichia coli* or* E. coli* is the preferred host system due to strong growth features and renowned physiology and metabolism [4]. Out of several different microbial PGA sources, PGA from* E. coli* (EcPGA) has outstanding potential for industrial purposes and also has been broadly studied as a part of academic research [7, 8]. Research focus on PGA production involving expression of “pac” gene from* E. coli* in* E. coli* has transcended diverse but linked areas like manipulation of the cell physiology [9–11], investigation of the effects of translocation efficiency [10, 11], and extracellular secretion [12, 13]. In addition, several studies have focused on expression of “pac” genes from other bacterial sources in* E. coli*. For a detailed review see the review by Srirangan et al. [4]. Along with* E. coli*, alternative hosts for expression have also been investigated: Gram positive* Bacillus* expression systems [14–17] and eukaryotic expression systems like* Saccharomyces cerevisiae* and* Pichia pastoris* [18–20].

Contrary to laboratory scale, production of PGA in large scales is challenging mainly due to this exclusive gene expression and various methods for protein maturation [4]. Therefore, it is mandatory to design key biochemical engineering approaches with improved upstream processing (strain manipulation), cultivation method, and highly robust downstream processing (DSP). The most important aspect that needs focus is the downstream processing with chromatography as an essential tool for PGA recuperation and refinement [21–24], direct immobilization without purification [25], and selective yield of the periplasmic part only and not the whole cell lysate [26, 27]. Very recently, a significant advancement has been achieved through extracellular production of PGA with high purity and yield has been reported in* E. coli* involving a single-step DSP which is based on tangential flow filtration anion-exchange membrane chromatography (TFF-AMEC) [28]. All these highlight the importance of application of a total quality system that needs to be built-in into the system rather than tested only on the end-products. It is our belief that all the distinct stages like strain manipulation, cultivation method, and purification can be significantly improved by implementing a quality control scheme by identifying the critical control points. Past study from our group has shown that a stringent quality system may be proposed for a general pilot scale production of any alternative and complementary medicine [29]. With this motivation we have analyzed and proposed a mechanism for inclusion of Six Sigma methodology in the production process. This may reduce batch-to-batch variations and produce better products that will adhere to the regulatory norms and meet quality issues.

## 2. Recent Progress in Enzymatic Transformation of Penicillin G to 6-APA

**Figure 1:**
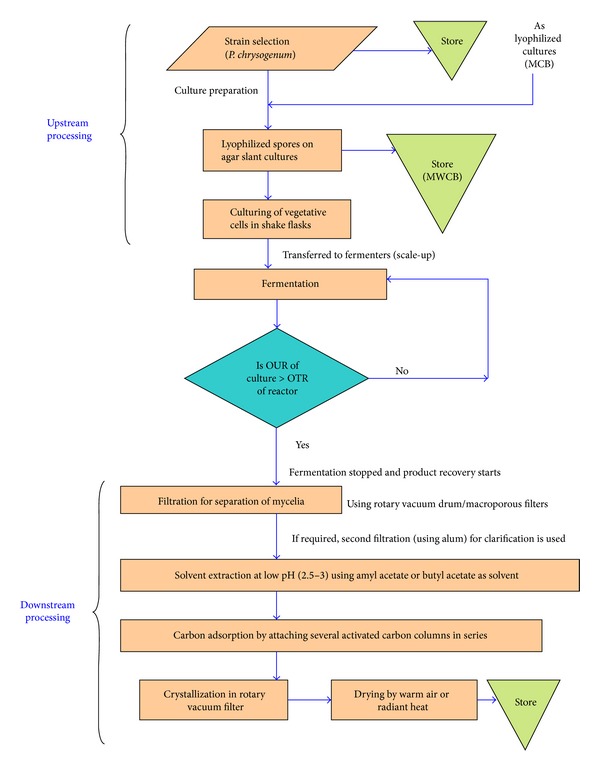
Schematic representation for large-scale production of Penicillin G (reproduced and redrawn from elsewhere [30]). Steps are self-explanatory. For a detailed account, see the source [30]. “OUR”: oxygen uptake rate, “OTR”: oxygen transfer rate, “MCB”: master cell bank, and “MWCB”: manufacturer's working cell bank.

Enzymatic hydrolysis of Penicillin G to obtain 6-APA is one of the most relevant examples of industrial implementation of biocatalysts [1, 31, 32]. 6-APA is the main precursor for the production of semisynthetic *β*-lactamic antibiotics [31]. A procedure for large-scale industrial production of Penicillin G is represented in Figure 1. 6-APA is produced at the scale of about 10,000 tons per year [33]. It has been reported that organic solvent mediated hydrolysis of Penicillin G, with the objective of extracting it from the culture medium, may greatly abridge the industrial manufacturing of 6-APA [34]. It may be noted that immobilization of enzyme on a carrier results in the loss of enzyme activity [35]. Therefore, in recent years, carrier-free immobilized enzymes such as cross-linked enzymes, cross-linked enzyme crystals (CLECs), and cross-linked enzyme aggregates (CLEAs) have been in regular use [36]. These procedures are beneficial over carrier-bound enzymes as the final preparation has high concentration of enzyme per unit volume [35]. Optimization of 6-APA manufacturing using cross-linked enzyme aggregates (CLEA) of* Bacillus badius* Penicillin G acylase (PAC) has also been reported [35]. According to the authors, this work has a strong potential for industrial application mainly because of faster conversion of Penicillin G to 6-APA by CLEA-PAC and also proficient reusability.

In a recent study, the enzymatic hydrolysis of Penicillin G for production of 6-APA was obtained by using Penicillin G acylase as catalyst in an aqueous-methyl isobutyl ketone (MIBK) system. According to the authors, the optimized conditions are initial pH 8.0, 5.0% (W = V) substrate (Penicillin G), temperature at 35°C, and the ratio of aqueous and organic phase 3 : 1 [33]. In a very recent study, different soil bacterial samples from different places of Dibrugarh (India) were isolated and incubated in presence of Penicillin G (2 mg/mL) for 48 hours at 37°C for conversion to 6-APA which was detected by thin layer chromatography (TLC) [37]. Also, the use of whole cell or isolated enzyme for the preparation of 6-APA has been reported [35, 38, 39]. This includes manufacturing of a fully efficient, permuted single-chain Penicillin G acylase [38], production of* Alcaligenes faecalis* Penicillin G acylase in* Bacillus subtilis* WB600 (pMA5) fed with partially hydrolyzed starch [39], and application of cross-linked enzyme aggregates of* Bacillus badius* Penicillin G acylase for the production of 6-APA [35]. In order to preserve high activity and specificity, better contamination control, and log life, various techniques have been used to immobilize the Penicillin G acylase either as free form or as whole cell enzyme [40]. However, it may be noted that further studies would benefit from optimizing the manufacturing of 6-APA using different immobilization techniques [37]. In the context of the current work, considering several options, we choose to base our hypothesis based on a recombinant* E. coli* based expression platform that must contain the “pac” gene from* E. coli* itself. Considering that once the upstream process is optimized, cultivation should be done with a high cell density and should be followed by extensive DSP, as suggested elsewhere [4, 34].

## 3. 6-APA: Precursor for Semisynthesis of ***β***-Lactam Antibiotics

In pharmaceutical industries, 6-aminopenicillanic acid (6-APA) is a very important intermediate product, primarily for being the precursor for the semisynthesis of *β*-lactam antibiotics [35] such as ampicillin and amoxicillin. Penicillin acylase is an enzyme produced by several bacteria and fungi. Although its metabolic role is not completely understood, it is widely used for the production of 6-APA. The main reason is that it can catalyze the hydrolysis of Penicillin G in this compound and phenylacetic acid [41]. This enzyme could also be used to catalyze the reverse reaction and the synthesis of the amide bond.

### 3.1. Mechanism of Action of Penicillin Acylase

6-APA is an integral *β*-lactam compound of various penicillins. Penicillins have a heterocyclic group consisting of a thiazolidine ring (with 5 members including one sulphur atom) joined with a *β*-lactam ring (with 4 members). These are distinguished from each other by the nature of the side chain, which is attached to the amine group in position 6 through a peptide link [42]. The fermentation of microbes of the* Penicillium chrysogenum* type makes it possible to obtain the greatest output of the corresponding penicillin. But there must be optimum culture conditions depending on the precursor used, which is normally PenG or PenV. Several other reactions also take place during this type of fermentation and alongside the penicillin biosynthesis reactions. By other reactions, we refer to the reactions forming other metabolites, as well as the degradation reactions of penicillin to penicillanic acid. They also include oxidation of the phenylacetic acid precursor to o-hydroxyphenylacetic acid (oxidized derivative). All these reactions must be controlled.

The resulting penicillin is converted into 6-APA by the reaction of removing the side chain linked to the amino group in position 6. Initially chemical processes achieved this reaction, but in recent times it is carried out through enzymatic processes using enzymes like penicillin acylase or amidase. The function of this enzyme is to recognize the side chain and break the peptide link joining it to the 6-APA nucleus. This reaction usually takes place in aqueous phase with a slightly alkaline pH. Subsequently, the 6-APA is extracted by adjusting the pH to the isoelectric point. The side chain component is first isolated through a solvent. This is followed by an additional aqueous-phase extraction, so that it may be recycled as a precursor in the fermentation itself. Of course, this has to be done following the appropriate tests. After all these, we can recover the 6-APA product, which is present in the crystallization mother liquors, through concentration by the reverse osmotic process. This is done at proper concentrations, which make crystallization easier [34].

## 4. Six Sigma Methodology: An Overview

### 4.1. Six Sigma: An Introduction

In statistics, we know that Sigma (*σ*) is a term that commonly represents standard deviation which is an indicator of the degree of deviation in a group of measurements or a process. Six Sigma (6*σ*) is a statistical perception that assesses a process in terms of faults or defects (see Table 1). It can be envisioned as a viewpoint of managing which centers on erasing defects through practices that stress understanding, measuring, and betterment processes [43]. Harry (1998) defines Six Sigma to be “a strategic initiative to boost profitability, increase market share and improve customer satisfaction through statistical tools that can lead to breakthrough quantum gains in quality” [44]. In 1987, Motorola first launched Six Sigma [43] and was soon followed by some leading electronic companies such as IBM, DEC, and Texas Instruments in early 1990s. In 1995, when GE and Allied Signal instigated Six Sigma as planned initiatives, a fast propagation took place in nonelectronic farms globally [45].

By detecting and eliminating the reasons of defects (errors) and minimizing inconsistency in production and business processes, Six Sigma helps to enhance the quality of process outputs. It crafts a unique infrastructure of personnel within the business organization (“Black Belts,” “Green Belts,” etc.) by employing an array of quality management methods. Each Six Sigma assignment that is carried out within an organization follows an established sequence of steps and has quantified economic targets [46].

### 4.2. Six Sigma: Principles

Six Sigma projects follow two project methodologies: DMAIC and DMADV. These have five phases each and are motivated by Deming's plan-do-check-act cycle [47]. While DMAIC is used for projects aimed at improving an existing business process, DMADV is used for projects aimed at creating new product or process designs [43]. Since in the current study we only deal with devising a strategy to improve the large-scale existing production of Penicillin G and its subsequent conversion to 6-APA, we will consider only DMAIC.

**Figure 2:**
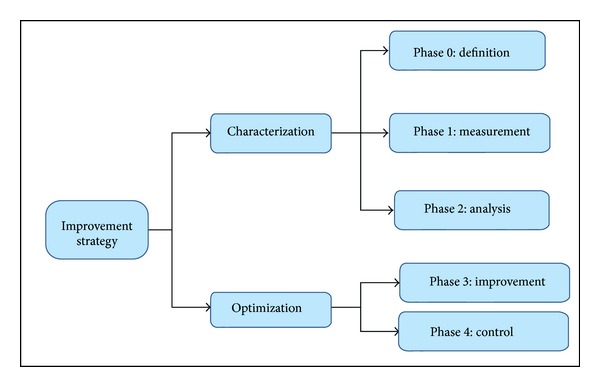
Improvement phases in the Six Sigma methodology (reproduced and redrawn from elsewhere [43]).

DMAIC (Define-Measure-Analyze-Improve-Control) process acts as a step forward policy and is probably the most important methodology in Six Sigma management [43]. This methodology allows genuine improvements and genuine results and works identically well on several entities (variation, cycle time, yield, design, etc.). It is divided into five phases as shown in Figure 2.

In each phase the major activities are as follows [43].* Phase 0 (Definition).* Here we identify the process or product that has to be improved. We may also standardize the principal product or process features of other leading global farms.* Phase 1 (Measurement).* Here, the product features such as reliant variables, relevant process mapping, essential measurement performing, result recording, and short- and long-term process capability estimating are selected. In identifying critical product features, quality function deployment (QFD) plays a significant role.* Phase 2 (Analysis).* Here we scrutinize and standardize the principal product/process performance metrics with different statistical and quality control tools. A gap analysis often ensues to detect the regular factors of thriving performance, that is, what factors explicate best-in-class performance.* Phase 3 (Improvement).* Here we identify those product performance features which have to be advanced to reach the target. This is followed by detecting the major sources of variation, identification of the principal process variables (by Taguchi methods and other vigorous experimental designs), and verification of the developed orders of principal process variables.* Phase 4 (Control).* The final phase is commenced by knowing that the new process conditions are documented and scrutinized via statistical process control (SPC) methods. Sometimes, it is wise to revisit one or more of the earlier phases depending upon the consequence of such a follow-on investigation.

### 4.3. Six Sigma Approach in Pharmaceutical Industries through Case Studies

As a part of the current work, it is essential to be acquainted with the current status of the application of total quality system for the production of pharmaceuticals, preferably through a few case studies. It is a probability that the modification of the fermentation unit of the bioprocess or improvement of operations in downstream processing may result in the design and optimization advancement of the pharmaceutical production process [48]. It is found that quality control strategies (including Six Sigma) as a means to either improve production or to enhance product quality in the pharmaceutical industries have been proposed or predicted through simulations by diverse groups [49–53]. An early study forecasted a theoretical model for penicillin synthesis, with qualitative prediction of different developments that are generally countered in experiments and also reviewed various models for secondary metabolite manufacturing [49]. This work was carried forward by Birol et al. [51] where the authors broadened the model by Bajpai and Reuss [49] and developed a simulation code for a fed-batch fermenter producing penicillin by including several additional parameters and trying to identify errors in a representative penicillin fermentation procedure. Both these studies throw some light on the possibility of minimal deviation from the ideal process flowchart that could be achieved through accurate modular predictions. Also, it is critical that any group embarking on such work comprehends the process well particularly while choosing correct output variables [52]. This has been shown by an earlier work where the operating pH and temperature turned out to be the decisive parameters that affected the penicillin yield [50].

Case studies that involve integration of Six Sigma methodology in pharmaceutical production unit have been conducted in related areas such as biological, parenteral operations, safety, and improved control of potency [53]. Six Sigma can serve as the driving force for continuous improvement by identifying the root cause or causes of low process yield, due to excessive variance in the desired specifications [52]. It is possible to advance the process controllability leading to enhanced sigma levels [54]. It has been proposed that the Six Sigma methodology may decrease discrepancy and focus on significant elements to attain continuous improvement by identifying and analyzing all of the constituent steps of prophylactic antibiotic administration and then observing them for improvement [55]. According to Nunnally and McConnell [53], the application of Six Sigma has helped in minimizing deviations in two of the key variables, pH and specific potency, in a fermentation set-up. Recently, a very interesting work has focused on an amalgamation of Six Sigma methodology with incorporated design and control and has predicted superior yield of archetypal penicillin [52]. This significant work, which used a combination of different quality control approaches including process analytical technologies (PAT) and Six Sigma methodology, demonstrated a 40% decrease in batch time, but simultaneously a considerable enhanced throughput yield and reduction in the concentration of contaminants. This facilitated our work which is based on similar motivation, but we have focused exclusively on Penicillin G and have extended the approach till its conversion to 6-APA.

## 5. Proposed Six Sigma Approach for Production of Penicillin G and Conversion to 6-APA

We are going to implement the five major principles of Six Sigma methodology (DMAIC) as indicated in Figure 2 and explained in Section 4. We will start with “DEFINE.” Here the approach is twofold: (a) DEFINE the process and (b) DEFINE the business case.

### 5.1. “DEFINE”

**Figure 3:**
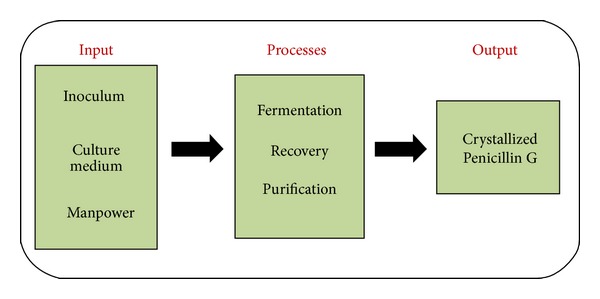
Schematic representation of producing crystallized Penicillin G.

*(a) “DEFINE” the Process.* The process of acquiring crystallized Penicillin G is represented in Figure 3. Briefly, the inputs consist of culture medium, inoculum (*Penicillium chrysogenum*), and trained personnel required to handle the operations and for process monitoring. Both fermentation and downstream processing (recovery and purification) have been integrated into processes for simplicity of the Six Sigma approach. The output is the crystallized form of Penicillin G.

*(b) “DEFINE” the Business Case*. Problem statement: product quality, safety, consistency, and purity depend on presence of contaminants. It is important to eliminate the contaminants comprehensively. Project goal: improvement of product quality, safety, consistency, and purity can be addressed by considering the following key issues: (A) examination of seed lots for endogenous and adventitious microbial contaminants (like retroviruses, bacteria, fungi, mycoplasma, etc.), done by some important tests: (i) reverse transcriptase (RT) assay and electron microscopy (EM) (for retroviruses), (ii) sterility and mycoplasma test,* in vitro* virus test, and genetic, immunological, and biochemical tests (for bacteria, mycoplasma, and fungi), and (iii) determination of inherent characteristics like isoenzyme pattern and chromosomal composition (for establishing cell identity), and (B) examination of cell and product stability during the production process.

In the past decade, several studies have stressed upon the fact that due to their extremely minute physical dimensions EM remains the preferred choice for visualization of most viruses (including retroviruses) either in liquid samples or in infected cells [56–59]. Though there have been some concerns raised over the usefulness of EM [60, 61], particularly when more sensitive methods such as the PCR and fluorescence light microscopy are available [57], the credibility of EM (particularly TEM) remains indispensible for some virological features, preliminary detection of unidentified viral agents and increased resolution for distinguishing between viral protein aggregates and viral particles with distinct structures, and is thus on the preferred list of regulatory agencies when inspecting for the viral safety of biological products [57]. In fact, we believe that inspection of pharmaceutical samples through a combination of an EM technique and other techniques like RT-PCR could be most potent for the identification of desired target. This combinatorial approach is highly beneficial for the analysis of complex biological systems (such as those involving retroviruses) because there is more data available to make valid conclusions possible than it would have been using a single approach [56–58]. Thus, in order to enforce a stringent quality control system, use of high-throughput analytical instruments and hyphenated techniques is necessary, even though this may significantly raise the expenditure and cost of implementation. While the authors acknowledge the fact that initial investment costs are high, especially those pertaining to EM, and research groups particularly in developing countries could be apprehensive in this regard, the possibility of enhanced grants from government agencies in helping procuring the instrument could be a source of hope.

### 5.2. “Measure” and “Analyze” the Current Process Performance

The objective of “Measure” is to identify critical measures in the penicillin production process that are necessary to evaluate the success which will meet the requirements of customer and to develop a methodology to collect data efficiently and to measure the process performance [43]. However, the most important part is to understand the elements of Six Sigma calculation and establish a threshold value for Sigma for the upstream, fermentation, and downstream processes. This is very important since it explains “how we are doing” all those implementations which we need to do. The main activities involved in this section are (i) identifying inputs (inoculums, media, etc.), processes (upstream, fermentation, and downstream processing), and output (6-APA, Penicillin G, etc.) indicators, (ii) developing operational definition and measurement plan, (iii) plotting and analyzing data, (iv) identifying any special cause, (v) constructing cause and effect matrix, and (vi) collecting other baseline performance data. To materialize what we have thought of, we need some good mold sampling techniques. From these, we may choose the one or two different techniques that suit best for* P. chrysogenum*. Some common mold sampling techniques [62] are described below.

#### 5.2.1. Air Sampling

To accumulate particles that are airborne, we need a tape, cassette, or a gathering device together with a regulated air pump [62]. Some common air-samplers are impaction samplers, cassette samplers, cassette-like samplers, Andersen samplers, and Andersen-type impaction samplers. Investigating the air may be helpful because of the huge supply source which has not yet been found in a building. This may be significant to analyze the relative molecule levels between an issue territory and a control region or in a prior zone and then afterward cleaning [62]. A qualitative analysis of an air sample by an expert technician can provide proof of a nearby problem mold reservoir in certain cases, for example, if the indoor sample contains long chains of* Penicillium* spores. Examining particles on a slide to count mold spores/cubic meter of air, mold spores/square meter of surface area, or mold CFU (colony-forming units) is a common practice in building investigations. These measures can be used to describe the results of some sampling or “mold testing” methods in the penicillin production plant. If there are more than 10000 molds present in one cubic meter of air in a building, the mold contamination is a reality, though this is not a customary quantitative standard [62].

#### 5.2.2. Tape Sampling

The inspected surface is first pressed with an agreeable cellophane tape, then evacuated, and altered to a clean surface for mailing to a lab. The tape is then readied by the lab for examination under microscope. The tape may also be extensively analyzed for identifying the genus and species of a particular mold. This is the minimum exorbitant gathering system accessible and is a favoured instrument [62]. In some cases genera determination alone is quite sufficient as some of the common problem-genera (*Penicillium* and* Aspergillus* spp.) do not have nonproblematic members that grow in buildings. If there are large amounts of molds present in a building, this is just a qualitative technique [62]. Combined with a visual inspection to locate target areas of risk and to find visible problems, it is the most essential component of a building mold investigation and is the method recommended by experts. When a tape is pressed into a sizeable area of anticipated mold growth on a surface, it collects majority of the material. This often includes enough structural material based on which persistent awkward mold genera and species may be isolated. When a sample is properly collected, it will definitely contain some significant tools that may aid in speciation, like hyphae, conidia, and so forth [62]. In short, we can say that the determination of the presence of a building mold problem (toxic or allergenic) versus cosmetic mold can usually be made from tape samples alone.

#### 5.2.3. Vacuum Sampling

For testing beddings, curtains, and so forth (which are soft goods) for enhanced levels of tainted spores, vacuum samples may be a suitable choice as a qualitative approach, which can be used as an inspection method for mold clearance. A vacuum or an air pump may be attached to a collection canister through which particles may be drawn onto the surface of the filter or into some particular gathering storage place [62]. The main R&D lab clears the filter onto a microscope slide, washes the filter onto a microscope slide, or uses another method to transfer particles for examination by microscope for preparation by culture.

#### 5.2.4. Swab Sampling

This is a good choice for microscopic inspection of the particles but may kill the identified hyphae and conidiophores [62]. We have to make use of swabs to sample for bacteriological contamination. Two principal ways of swab processing are either by culturing (the swab is rolled across a culture plate for culturing and subsequent sample detection) or by a direct examination (similar to tape sampling explained above) [62]. Please note that techniques such as amplification through polymerase chain reaction or PCR may also be tried for the detection of individual species and/or genus with superior precision and fairly rapidly. The method requires costly equipment and is not available at most laboratories. Perhaps more important is that the database of PCR identification information is limited to a small set of species compared with the wide range of genera/species which normally occur. The most commonly used seed lot system involves one 100–200 vial master cell bank (MCB) and one or more 400–500 vial manufacturer's working cell banks (MWCB) derived from the MCB. While the adventitious agents, such as mycoplasma, viruses, and prions can be initiated during the handling procedure or may be added by the parental cells and nutrients and can be identified via specific tests such as sterility and mycoplasma tests, the contaminants of bacterial origin are usually identified by cell substrate inoculation in suitable microbial media and incubating for 2–4 weeks followed by biochemical tests and microscopic examination [63]. Mycoplasma is detected by DNA staining or immunological tests.* Inoculum stability* is also measured at three levels: (i) during the storage of cell banks, (ii) through several cell divisions in each production batch, and (iii) during various production batches. Suspension systems, such as stirred tank or airlift reactors, and entrapped systems, such as fluidized bed or hollow fiber reactors, are commonly used for Penicillin G production. Stirred tank and airlift reactors can operate in batch and fed-batch mode.

### 5.3. “Improve” the Process

*(A) In-Process Testing*. In-process testing must be done at 3 different stages. They are (i) testing of samples collected at the preculture (inoculum) step, (ii) testing of samples collected at specific intervals during the large-scale cultivation process, and (iii) testing of samples collected at the end of the bioreactor run. The technique is essential to distinguish novel or triggered foreign agents. There must be a methodical exploration in order to resolve the cause of contamination and widespread decontamination of the bioreactor and all the affected areas are recommended. Also, the entire analysis and decontamination actions must be properly documented and preserved.

*(B) Culture Stability Testing*. Factors such as specific productivity (pg/cell/day), cell growth kinetics, and the portion of producer cells in the cell bank (clonality) may assist in scrutinizing the similarity between the MCB and MWCB. The manufacturing process may witness the subsequent stage of cell line stability tests being performed, for which the cells are assembled from initial, middle, and terminal phases of the cultivation cycle [63]. For example, in a batch process, cells are collected from the preculture, inoculum, and three times during production: the lag phase, the exponential growth phase, and at the end of production.

### 5.4. “Controls” to Hold the Gains

Several sophisticated supervising and organizing strategies, for instance, optical density probes and image analysis attached to some superior controls system have been applied in small-scale fermentation [63]. However, these advanced strategies are at most cautiously applied in large-scale fermentation processes. Production processes typically utilize online control for pH, temperature, and dissolved oxygen only. Batch cultures often require little to no additional monitoring and control other than frequent offline measurements of cell density and viability [43]. Fed-batch cultures require control of nutrient feed rates. Control strategies based on cell growth estimates, online oxygen utilization rates (OUR), or glucose and lactate measurements have been applied. However, it is a common observation that, in large-scale processes, the rates for addition of nutrients are more often based on time generated proceedings and utilize the control for feedback less frequently [63]. Process validation demonstrates that the production process performs as intended in a consistent manner. It must be demonstrated that the purification process will remove known impurities such as DNA and medium components. The information for approval is ordinarily assembled by studying full-scale assembling runs and likewise by “challenge" studies utilizing model viruses, DNA, and process contaminants on scaled-down variants of full-scale process. The itemized dissection of a little number of full-scale assembling runs can give immediate confirmation that the refinement procedure can reliably evacuate known polluting influences for which delicate examinations exist [63].

## 6. Conclusions

Though the entire study is hypothetical, we have been able to identify some critical points which may act as potential sites for integrating the Six Sigma methodology. First the process and the business case need to be defined separately which could be trailed by investigation of the seed lots and the cell or culture stability. Process characterization follows as an integral part of measure and analysis. All the potential adventitious agents must be identified using scientific tests and provisions must be included to get rid of them. All the fermentation parameters may be optimized at this point. Also, process improvement needs constant attention by in-process testing and inspection of the culture stability. This is followed by validation. It is critical therefore that the process works as intended for an infinite period. We have also considered several strategies for process validation and tried to correlate “DMAIC” with respect to Penicillin G production. It is our understanding that Six Sigma can be effectively used as a part of total quality system for the mass production of Penicillin G and its subsequent conversion to 6-APA.

## Figures and Tables

**Table 1:** Probability of defects of different Sigma levels at a fixed process mean reproduced from elsewhere [43].

Sigma quality level	Nondefect rate	Defect rate (ppm)
*σ*	68.26894	317,311
2*σ*	95.44998	45,500
3*σ*	99.73002	2,700
4*σ*	99.99366	63.4
5*σ*	99.999943	0.57
6*σ*	99.9999998	0.002

“ppm”: parts per million.

## References

[1] Chandel AK, Rao LV, Narasu ML, Singh OV (2008). The realm of penicillin G acylase in *β*-lactam antibiotics. *Enzyme and Microbial Technology*.

[2] Parmar A, Kumar H, Marwaha SS, Kennedy JF (2000). Advances in enzymatic transformation of penicillins to 6-aminopenicillanic acid (6-APA). *Biotechnology Advances*.

[3] Peñalva MA, Rowlands RT, Turner G (1998). The optimization of penicillin biosynthesis in fungi. *Trends in Biotechnology*.

[4] Srirangan K, Orr V, Akawi L, Westbrook A, Moo-Young M, Chou CP (2013). Biotechnological advances on penicillin G acylase: pharmaceutical implications, unique expression mechanism and production strategies. *Biotechnology Advances*.

[5] Bush K, Chadwick DJ, Goode J (2007). The evolution of *β*-lactamases. *Antibiotic Resistance: Origins, Evolution, Selection and Spread*.

[6] Brugging A, Roos EC, de Vroom E (1998). Penicillin acylase in the industrial production of *β*-lactam antibiotics. *Organic Process Research and Development*.

[7] Karthikeyan R, Surianarayanan M, Sudharshan S, Gunasekaran P, Asit Baran M (2011). Biocalorimetric and respirometric studies on production of penicillin G acylase from Bacillus badius pac in *E. coli* DH5*α*. *Biochemical Engineering Journal*.

[8] Sudhakaran VK, Deshpande BS, Ambedkar SS, Shewale JG (1992). Molecular aspects of penicillin and cephalosporin acylases. *Process Biochemistry*.

[9] Chou CP (2007). Engineering cell physiology to enhance recombinant protein production in *Escherichia coli*. *Applied Microbiology and Biotechnology*.

[10] Scherrer S, Robas N, Zouheiry H, Branlant G, Branlant C (1994). Periplasmic aggregation limits the proteolytic maturation of the *Escherichia coli* penicillin G amidase precursor polypeptide. *Applied Microbiology and Biotechnology*.

[11] Sriubolmas N, Panbangred W, Sriurairatana S, Meevootisom V (1997). Localization and characterization of inclusion bodies in recombinant *Escherichia coli* cells overproducing penicillin G acylase. *Applied Microbiology and Biotechnology*.

[12] Choi JH, Lee SY (2004). Secretory and extracellular production of recombinant proteins using *Escherichia coli*. *Applied Microbiology and Biotechnology*.

[13] Gumpert J, Cron H, Plapp R, Niersbach H, Hoischen C (1996). Synthesis and secretion of recombinant penicillin G acylase in bacterial L-forms. *Journal of Basic Microbiology*.

[14] Westers L, Westers H, Quax WJ (2004). *Bacillus subtilis* as cell factory for pharmaceutical proteins: a biotechnological approach to optimize the host organism. *Biochimica et Biophysica Acta*.

[15] Rajendhran J, Krishnakumar V, Gunasekaran P (2003). Production of penicillin G acylase from Bacillus sp.: effect of medium components. *World Journal of Microbiology and Biotechnology*.

[16] Yang S, Huang H, Zhang R, Huang X, Li S, Yuan Z (2001). Expression and purification of extracellular penicillin G acylase in *Bacillus subtilis*. *Protein Expression and Purification*.

[17] Yang Y, Biedendieck R, Wang W (2006). High yield recombinant penicillin G amidase production and export into the growth medium using Bacillus megaterium. *Microbial Cell Factories*.

[18] Cregg JM, Cereghino JL, Shi J, Higgins DR (2000). Recombinant protein expression in Pichia pastoris. *Applied Biochemistry and Biotechnology B*.

[19] Marešová H, Marková Z, Valešová R, Sklenář J, Kyslík P (2010). Heterologous expression of leader-less pga gene in Pichia pastoris: intracellular production of prokaryotic enzyme. *BMC Biotechnology*.

[20] Mattanovich D, Branduardi P, Dato L, Gasser B, Sauer M, Porro D (2012). Recombinant protein production in yeasts. *Recombinant Gene Expression*.

[21] Aguilar O, Albiter V, Serrano-Carreón L, Rito-Palomares M (2006). Direct comparison between ion-exchange chromatography and aqueous two-phase processes for the partial purification of penicillin acylase produced by *E. coli*. *Journal of Chromatography B*.

[22] Fonseca LP, Cabral JMS (2002). An integrated downstream processing strategy for the recovery and partial purification of penicillin acylase from crude media. *Journal of Chemical Technology and Biotechnology*.

[23] Liu Y-C, ChangChien C-C, Suen S-Y (2003). Purification of penicillin G acylase using immobilized metal affinity membranes. *Journal of Chromatography B*.

[24] Sudhakaran VK, Shewale JG (1987). Hydrophobic interaction chromatography of penicillin amidase. *Biotechnology Letters*.

[25] Chen C-I, Ko Y-M, Shieh C-J, Liu Y-C (2011). Direct penicillin G acylase immobilization by using the self-prepared immobilized metal affinity membrane. *Journal of Membrane Science*.

[26] de León A, García B, Barba de la Rosa AP, Villaseñor F, Estrada A, López-Revilla R (2003). Periplasmic penicillin G acylase activity in recombinant *Escherichia coli* cells permeabilized with organic solvents. *Process Biochemistry*.

[27] Rodriguez M, Güereca L, Valle F, Quintero R, López-Munguia A (1992). Penicillin acylase extraction by osmotic shock. *Process Biochemistry*.

[28] Orr V, Scharer J, Moo-Young M (2012). Simultaneous clarification of *Escherichia coli* culture and purification of extracellularly produced penicillin G acylase using tangential flow filtration and anion-exchange membrane chromatography (TFF-AEMC). *Journal of Chromatography B*.

[29] Pan S, Neeraj A, Srivastava KS, Kishore P, Danquah MK, Sarethy IP (2013). A proposal for a quality system for herbal products. *Journal of Pharmaceutical Sciences*.

[30] Shuler ML, Kargi F (2002). *Bioprocess Engineering—Basic Concepts*.

[31] van de Sandt EJAX, de Vroom E (2000). Innovations in cephalosporin and penicillin production: painting the antibiotics industry green. *Chimica Oggi*.

[32] Arroyo M, de la Mata I, Acebal C, Castillón MP (2003). Biotechnological applications of penicillin acylases: state-of-the-art. *Applied Microbiology and Biotechnology*.

[33] Fang S-G, Qiang T, Liu R-J, Xu X-M, Zhang Y-W (2010). Enhanced production of 6-aminopenicillanic acid in aqueous methyl isobutyl ketone system with immobilized penicillin G acylase. *Preparative Biochemistry and Biotechnology*.

[34] Abian O, Mateo C, Fernández-Lorente G, Guisán JM, Fernández-Lafuente R (2003). Improving the industrial production of 6- APA: enzymatic hydrolysis of penicillin G in the presence of organic solvents. *Biotechnology Progress*.

[35] Rajendhran J, Gunasekaran P (2007). Application of cross-linked enzyme aggregates of Bacillus badius penicillin G acylase for the production of 6-aminopenicillanic acid. *Letters in Applied Microbiology*.

[36] Cao L, van Langen L, Sheldon RA (2003). Immobilised enzymes: carrier-bound or carrier-free?. *Current Opinion in Biotechnology*.

[37] Dolui AK, Sahana S, Kumar A (2012). Studies on production of 6-aminopenicillanic acid by free and *κ*-carrageenan immobilized soil bacteria. *Indian Journal of Pharmaceutical Education and Research*.

[38] Flores G, Soberón X, Osuna J (2004). Production of a fully functional, permuted single-chain penicillin G acylase. *Protein Science*.

[39] Zhang M, Shi M, Zhou Z, Yang S, Yuan Z, Ye Q (2006). Production of *Alcaligenes faecalis* penicillin G acylase in *Bacillus subtilis* WB600 (pMA5) fed with partially hydrolyzed starch. *Enzyme and Microbial Technology*.

[40] Norouzian D, Javadpour S, Moazami N, Akbarzadeh A (2002). Immobilization of whole cell penicillin G acylase in open pore gelatin matrix. *Enzyme and Microbial Technology*.

[41] Xue-Jun C, Xing-Yan W, Fonseca LJP, Cabral JMS, Marcos JC (2004). Production of 6-aminopenicillanic acid in aqueous two-phase systems by recombinant *Escherichia coli* with intracellular penicillin acylase. *Biotechnology Letters*.

[42] Ruiz MO, Yecora NF, de Prado EG, Alba AV, Maldonado FS Process for producing 6-amino-penicillanic acid and phenylacetic acid.

[43] Park SH (2003). *Six Sigma for Quality and Productivity Promotion*.

[44] Harry MJ (1998). *The Vision of Six Sigma*.

[45] Hendricks A, Kelbaugh RL (1998). Implementing Six Sigma at GE. *The Journal of Quality and Participation*.

[46] Antony J Pros and cons of Six Sigma: an academic perspective. http://web.archive.org/web/20080723015058/http://www.onesixsigma.com/node/7630.

[47] de Feo JA, Barnard WW (2005). *Juran Institute's Six Sigma Breakthrough and Beyond: Quality Performance Breakthrough Methods*.

[48] Bogle IDL, Cockshott AR, Bulmer M, Thornhill N, Gregory M, Dehghani M (1996). A process systems engineering view of biochemical process operations. *Computers and Chemical Engineering*.

[49] Bajpai RK, Reuss M (1980). A mechanistic model for penicillin production. *Journal of Chemical Technology and Biotechnology*.

[50] Kheirolomoom A, Kazemi-Vaysari A, Ardjmand M, Baradar-Khoshfetrat A (1999). The combined effects of pH and temperature on penicillin G decomposition and its stability modeling. *Process Biochemistry*.

[51] Birol G, Ündey C, Çinar A (2002). A modular simulation package for fed-batch fermentation: penicillin production. *Computers and Chemical Engineering*.

[52] Dassau E, Zadok I, Lewin DR (2006). Combining Six-Sigma with integrated design and control for yield enhancement in bioprocessing. *Industrial and Engineering Chemistry Research*.

[53] Nunnally K, McConnell JS (2007). *Six Sigma in the Pharmaceutical Industry: Understanding, Reducing, and Controlling Variation in Pharmaceuticals and Biologics*.

[54] Seider WD, Seader JD, Lewin DR (2004). *Product and Process Design Principles: Synthesis, Analysis, and Evaluation*.

[55] Gordon SM (2006). Antibiotic prophylaxis against postoperative wound infections. *Cleveland Clinic Journal of Medicine*.

[56] Reid GG, Milne EW, Coggins LW, Wilson NJ, Smith KT, Shepherd AJ (2003). Comparison of electron microscopic techniques for enumeration of endogenous retrovirus in mouse and Chinese hamster cell lines used for production of biologics. *Journal of Virological Methods*.

[57] Roingeard P (2008). Viral detection by electron microscopy: past, present and future. *Biology of the Cell*.

[58] Bharat TAM, Davey NE, Ulbrich P (2012). Structure of the immature retroviral capsid at 8Å resolution by cryo-electron microscopy. *Nature*.

[59] Roberts A, Thorley BR, Bruggink LD, Marshall JA (2013). Electron microscope detection of an endogenous infection of retrovirus-like particles in L20B cells. *Journal of Electron Microscopy*.

[60] Curry A, Bryden A, Morgan-Capner P (1999). A rationalised virological electron microscope specimen testing policy. *Journal of Clinical Pathology*.

[61] McCaughey C, O’Neill HJ, Wyatt DE (2000). Rationalised virological electron microscope specimen testing policy. *Journal of Clinical Pathology*.

[62] Friedman D Introduction to mold testing and sampling. http://inspectapedia.com/mold/Mold_Test_Method_Validity.htm.

[63] Harris WJ, Adair JR (1997). *Antibody Therapeutics*.

